# Improved Expression of Recombinant Human Factor IX by Co-expression of GGCX, VKOR and Furin

**DOI:** 10.1007/s10930-014-9550-5

**Published:** 2014-02-25

**Authors:** Jianming Liu, Anna Jonebring, Jonas Hagström, Ann-Christin Nyström, Ann Lövgren

**Affiliations:** 1 Discovery Sciences, AstraZeneca R&D Mölndal, 431 83 Mölndal, Sweden; 2 CVMD iMed, AstraZeneca R&D Mölndal, 431 83 Mölndal, Sweden

**Keywords:** Recombinant factor IX, Haemophilia, Co-expression, Purification, γ-Carboxylation, CHO cells

## Abstract

Recombinant human FIX concentrates (rhFIX) are essential in the treatment and prevention of bleeding in the bleeding disorder haemophilia B. However, due to the complex nature of FIX production yields are low which leads to high treatment costs. Here we report the production of rhFIX with substantially higher yield by co-expressing human FIX with GGCX (γ-glutamyl carboxylase), VKOR (vitamin K epoxide reductase) and furin (paired basic amino acid cleaving enzyme) in Chinese hamster ovary (CHO) cells. Our results show that controlled co-expression of GGCX with FIX is critical to obtain high rhFIX titre, and, that co-expression of VKOR further increased the yield of active rhFIX. Furin co-expression improved processing of the leader peptide of rhFIX but had a minor effect on yield of active rhFIX. The optimal expression level of GGCX was surprisingly low and required unusual engineering of expression vector elements. For VKOR and furin the control of expression was less critical and could be achieved by standard vector element. Using our expression vectors an rhFIX-producing clone with an expression level of up to 30 mg/L of active rhFIX was obtained. In addition an efficient single step purification method was developed to obtain pure and active rhFIX with up to 94 % yield.

## Introduction

Coagulation factor IX (FIX) is a 55 kDa plasma protein that has an essential role in blood coagulation. Deficiency of FIX leads to haemophilia B, which is a bleeding disorder characterized by impaired coagulation and increased tendency to bleed. Haemophilia B is caused by mutations affecting the X chromosome linked FIX gene, and occurs in 1 of 30,000 male births [1, 2]. In haemophilia B patients, bleedings are typically recurrent and often affect the knees, ankles, and elbows. Complications include haemophilic arthropathy, resulting in damaged, painful joints and restricted mobility [3]. With the exception of patients with mild forms of the disease, treatment or prevention of bleeding episodes in haemophilia B patients is based on the injection of coagulation factor IX concentrate [4, 5]. The replacement coagulation factors are typically obtained from pooled human plasma or from recombinant preparations [3]. Administration of proteins purified from plasma confers a risk for transmission of infectious agents, and, the supply of suitable raw material is limited. Production of recombinant human FIX (rhFIX) for medical use is however not a simple task, and there is currently only one product on the market, Benefix [6]. Therefore, the availability of safe and cost-effective rhFIX concentrates still constitutes an unmet medical need.

FIX is synthesized in liver as a zymogen which is converted to a serine protease after activation in plasma. Before it is secreted into plasma, FIX undergoes several complex post-translation modifications (PTMs), including γ-carboxylation of 12 N-terminal glutamic acid residues, N- and O-linked glycosylation, phosphorylation, β-hydroxylation, sulfation, disulfide bond formation, and proteolytic processing of the signal peptide and the propeptide. Mature FIX consists of four distinct domains: the N-terminal Gla-domain, the EGF-like domain, the activating peptide (AP) domain and the C-terminal serine protease domain. Among the required PTMs is carboxylation of glutamic acid residues to form γ-glutamic acid (Gla) in the Gla domain, which is a major challenge in obtaining high-titer expression of fully functional rhFIX [7]. In this paper we describe engineering of a Chinese hamster ovary (CHO) cell line by introduction of three additional modifying enzymes supporting γ-carboxylation and proteolytic processing of FIX, resulting in dramatically improved productivity of fully functional rhFIX.

## Materials and Methods

### Materials

CHO-S cells, cell culture media including CD-CHO medium, G418, Lipofectamine 2000, non-essential amino acids supplement, HT supplement, human liver cDNA, mammalian vector pcDNA3.1V5/His, pcDNA3.1Hygro, pcDNA3.1+, pZeoSV2+, Pfx DNA polymerase, T4 polynucleotide kinase were purchased from Invitrogen. Konakion^®^ from Roche was used as a source of vitamin K. Oligonucleotide DNA fragments, formic acid, Tris–HCl, dithiothreitol and iodoacetamide were purchased from Sigma-Aldrich and acetonitrile was from Fisher Chemical. Trypsin was purchased from Promega and Arg-C and Lys-C were from Roche Diagnostics. ZYMUTEST Factor IX ELISA kit and BIOPHEN Factor IX assay kit were from HYPHEN BioMed. ROX Factor IX activity assay kit was from Rossix (Mölndal, Sweden).

### Construction of Expression Vectors

All cloning methods were according to standard methods and/or manufacturers’ recommended procedures.

#### NopA and hglx Cloning Vectors

DNA encoding human GGCX was amplified from liver cDNA using primers hglx5 (5′-TCCGCAGAGCAATGGCGGTGTCT-3′) and hglx3 (5′-CCAACATCTGGCCCCTTCAGAACT-3′). The GGCX PCR product was first cloned into the TA-TOPO treated vector pcDNA3.1V5/His (Invitrogen). GGCX encoding cDNA under the control of the SV40 promoter was obtained by transfer of the GGCX encoding fragment from pcDNA3.1V5/His TA-TOPO to the pZeoSV2+ vector, using restriction enzymes *Bam*H1 and *Not*I. The *EcoR*V-*Not*I restriction sites downstream of the GGCX insert were removed. A blunted *Cla*I-*Bcl*I fragment from the resulting pZeoSV2-GGCX plasmid (containing the SV40 promoter and the GGCX containing insert, but not the polyadenylation site and polyadenylation signal downstream of the GGCX encoding sequence) was then cloned into the blunted *Dra*III restriction site of pcDNA3.1+. A clone with the pSV40-GGCX fragment inserted in tandem (same transcriptional direction) relative to the CMV promoter was selected (pNopA). The *hglx* vector was constructed similar as the NopA vector, but in order to give higher GGCX levels the polyadenylation signal from pZeoSV2+ was included in the pSV40-GGCX-pA fragment cloned into the blunted *Dra*III site of pcDNA3.1, and, a clone with the GGCX-containing fragment in the reverse order compared to the NopA vector was selected.

#### FIX Expression Vectors F9*NopA* and F9*hglx*

Human coagulation factor IX cDNA (GeneBank NM_000133) was amplified from human Gene Pool liver cDNA (Invitrogen) using oligonucleotide primers F9f.ampl (5′-CACCATGCAGCGCGTGAACATGAT-3′) and F9r.ampl (5′-CCTTGGAAATCCATCTTTCATTA-3′). The human FIX fragment was PCR amplified using Pfx DNA polymerase. The blunt-ended PCR product was phosphorylated using T4 polynucleotide kinase, and cloned into the *EcoR*V digested and de-phosphorylated pcDNA-GGCX vectors NopA and hglx, to give F9NopA and F9hglx, respectively. The final expression vector sequences are available from GenBank under accession numbers JA237977 for F9hglx and JA237976 for F9NopA.

#### Vector VKORhygro

The VKOR coding sequence was PCR amplified from human liver cDNA (Clontech) using primers: VF1 (5′-CACCATGGGCAGCACCTGGGGGA-3′) and VR1 (5′-GCTCAGTGCCTCTTAGCCTT-3′) and TA-TOPO cloned into pcDNA3.1-V5/His (Invitrogen). The VKOR coding sequence was released by *Hind*III and *Not*I and then subcloned into the pcDNA3.1Hygro vector using the same restriction enzymes to give construct pVKORhygro (GenBank accession DM079693).

#### Vector pZeoSV2-Furin

The human furin/PACE coding sequence was cloned into pZeoSv2(+) vector (Invitrogen) using the *Afl*II and *EcoR*V restriction sites to obtain pZeoSv2-Furin (GenBank KF886270).

The correct coding sequences of FIX, GGCX, VKOR and furin were confirmed by DNA sequencing.

### Human FIX Cell Line Development

CHO-S cells were cultured in DMEM F12 with GlutaMAX and 10 % FBS, and transfected with linearized F9NopA or F9hglx according to recommendations from Invitrogen. Cells were seeded into 96-well plates with selective medium containing G418 the day after transfection. Screening for rhFIX expression was done by ELISA. The best FIX producing clone pool was subjected to limiting dilution cloning. During expansion clones were repeatedly screened for rhFIX expression using an ELISA. Three clones per construct were selected and adapted to growth in serum free medium. After adaption, clones were evaluated and compared based on growth properties and rhFIX expression level (using both ELISA and Western blot analysis). The best clone N4D5 was found from the F9NopA transfected cells. Clone N4D5 was then transfected with the VKORhygro construct, linearized with *Ssp*I, and hygromycin resistant clones were selected and screened for improved productivity with both FIX activity assay and ELISA. After limiting dilution cloning and test of productivity the clone A2F8 was chosen for further studies.

The A2F8 cell line was transfected with pZeoSv2-Furin, linearized by restriction enzyme *Ssp*I. After Zeocin selection, stable oligo-clonal pools were screened for improved FIX productivity using a chromogenic FIX assay (ROX-FIX) and clone 2F6 was selected and cryo-preserved in CD-CHO medium with 5 % DMSO.

### Quantification of rhFIX and GGCX mRNA Expression

Recombinant hFIX-producing clones were grown at 32–37 °C, in 100 mL protein free chemically defined medium supplemented with vitamin K. Samples of 5–10 mL were collected at peak rhFIX concentration and RNA was isolated with Trizol™ according to the protocol supplied by the vendor (Invitrogen). The isolated RNA was DNase I treated with the kit DNA-free™ from Ambion. cDNA synthesis was carried out using hexamers primers and kit contents from Superscript™ First-Strand Synthesis System for RT-PCR (Invitrogen). The RNA was analyzed for content of human FIX and human GGCX transcripts, as well as transcripts of the GAPDH control (house-keeping) gene, which was used to allow comparison of different samples.

Primers for rhFIX were:

- Forward primer; 5′-AATAGTGCTGATAACAAGGTGGTTTG-3′,
- Reverse primer; 5′-CACTGCTGGTTCACAGGACTTCT-3′ and
- Probe; 5′-TCCTGTACTGAGGGATATCGACTTGCAGAAAAC-3′ (Amplicon length 84 bp).

Human GGCX primers were:

- Forward primer; 5′-ACACCTCTGGTTCAGACCTTTCTT-3′,
- Reverse primer; 5′-AATCGCTCATGGAAAGGAGTATTT-3′ and
- Probe; 5′-CAACAAAGGCTCCAGGAGATTGAACGC-3′ (Amplicon length 86 bp)

Primers were manufactured by Operon/Qiagen and the probes were ordered from Applied Biosystems. Rodent GAPDH control primers and probe were also used (Applied Biosystems; ABI # 4308318 TaqMan^®^ Rodent GAPDH Control Reagents Protocol)-Amplicon length 177 bp. The Real-Time RT-PCR reactions were performed on the ABI Prism™ 7700 Sequence detector, Applied Biosystems. The expected length of the amplified PCR products was confirmed on agarose gels.

Messenger RNA levels were found to peak at different days depending on culture temperature and culture inoculum size, but they were found to correspond well with peak concentration of rhFIX in the culture medium.

### Expression and Purification of rhFIX

Cells were inoculated into fresh CD-CHO culture medium, supplemented with 1× HT, 1× Glutamax, 1× NEAA and 0.5 μg/mL vitamin K. Cells were cultured to a density of 5.3 × 10^6^ cells/mL in shake flasks incubated at 37 °C, 10 % CO_2_ and 150 rpm). Medium was then switched to an in-house developed production medium and cell density adjusted to 4 × 10^6^ cells/mL. Recombinant hFIX was expressed for 4 days at 32 °C, 10 % CO_2_ before harvest. The harvested culture medium was concentrated and buffer exchanged to Buffer A0 (25 mM Hepes, 0.4 % Na-Citrate, 50 mM NaCl, pH 6.8). The sample was loaded onto a 5 mL HiTrap Q FF column (GE Healthcare, Sweden) pre-equilibrated with Buffer A0 using Äkta FPLC (GE Healthcare, Sweden). After loading, the column was rinsed with buffer A0 first, then it was washed with Buffer A1 (25 mM Tris–HCl, 0.1 M NaCl, pH 8.0) followed by Buffer A2 (25 mM Tris–HCl, 0.2 M NaCl, pH 8.0). The rhFIX protein was eluted from the column with Buffer B1 (25 mM Tris–HCl, 0.1 M NaCl, 20 mM CaCl_2_, pH 8.0). The flow rate was kept at 4 mL/min during the purification process. Fractions were analyzed on SDS-PAGE gels, and fractions containing pure rhFIX protein were pooled and stored at −80 °C.

### Human FIX ELISA Assay

Recombinant human FIX protein in cell culture media was determined by using ZYMUTEST Factor IX ELISA kit according to the manufacturer’s protocol (HYPHEN BioMed, France). Purified human FIX (Haematologic Technologies Inc. Essex Junction, Vermont) or BeneFIX (Wyeth) was used as a standard.

### FIX Activity Assays

Recombinant FIX activity was determined in a chromogenic assay using BIOPHEN Factor IX assay kit from HYPHEN BioMed (France, Ref 221802) or ROX Factor IX activity assay (Rossix, Mölndal, Sweden) according to the manufacture’s protocol. Commercial recombinant human FIX (BeneFIX, Wyeth) or purified human FIX from Haematologic Technologies Inc. (Essex Junction, Vermont) was used as a standard in the activity assays. At early stages FIX clot assay (American Diagnostica Inc., Stamford, Conneticut) was used, but was later replaced with the chromogenic assays due to the convenience and higher sensitivity of these assays.

### SDS-PAGE and Western Blot Analysis

Purified recombinant FIX was analyzed on a 10 % Bis–Tris SDS-PAGE gel under reducing condition, and the gel was stained with InstantBlue solution (Expedeon, UK). The cell culture media samples were run on 10 % SDS-PAGE gels, proteins were transferred to a PVDF membrane according to the standard method and FIX protein on the blot was detected using a polyclonal rabbit anti-serum raised against FIX purified from human plasma.

### Mass Spectrometry Analysis

All LC/MS analyses were performed on an Agilent 1200 Series HPLC-Chip interfaced to an Agilent 6520 Q-TOF mass spectrometer. Chromatography was performed on an HPLC-Chip that had a 40 nL enrichment column and a 43 mm × 75 μm analytical column packed with ZORBAX 300SB, 5 μm particles. A C18 column was used for peptide analysis and the full-length protein was analyzed using a C8 column. Samples were loaded onto the enrichment column at a flow rate of 4 μL/min with Eluent A (97.5 % water, 2.5 % acetonitrile and 0.1 % formic acid). Elution was performed from the analytical column using a gradient from 5 % Eluent B (95 % acetonitrile, 5 % water and 0.1 % formic acid) to 65 % B in 20 min, followed by a step gradient to 95 % B before equilibration with 5 % B. The flow rate used during separation was 0.3 μL/min.

Before peptide mapping the cysteines were reduced using 10 mM dithiothreitol at 95 °C for 5 min and allowed to cool down to RT followed by alkylation with 55 mM iodoacetamide in the dark for 60 min. Proteins were digested with trypsin, Arg-C or Lys-C at 37 °C with an enzyme-to-substrate ratio (w/w) of 1:20 overnight according to descriptions given by the manufacturers. The peptide solutions were subjected to HPLC-Chip-MS/MS analysis. The full-length protein was diluted with 0.1 % formic acid in water to a final concentration of 1 pmol/μL and subjected to HPLC-Chip-MS analysis.

### γ-Carboxylation Analysis of Glutamic Acid Residues

In this study the content of γ-carboxylated glutamic acid residues was examined using peptide mapping and mass spectrometry. After alkylation of the cysteines and treatment with endoproteinase Lys-C of rhFIX, the N-terminal amino acid sequence 1–43 was formed. This amino acid sequence contains all 12 potential glutamic acid residues available for γ-carboxylation in FIX. The 1–43 peptide contained different numbers of γ-carboxylated glutamic acid residues. When trypsin or endoproteinase Arg-C were used as proteases there was information about the content of γ-carboxylated glutamic acid in position Glu-40.

## Results

### Generation of Stable rhFIX Cell Line

**Fig. 1:**
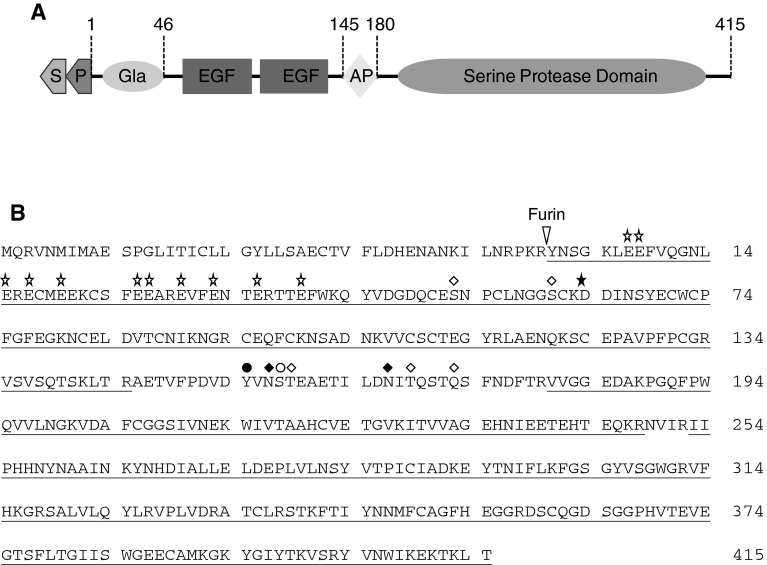
Schematic presentation of human FIX domain structure and its amino acid sequence. *Arabic numbers* 1–415 indicate amino acid positions in the mature FIX protein. **a** FIX domain structure. The signal peptide (S) and the propeptide (P) are removed to form mature FIX. In the mature part of FIX the γ-carboxy glutamic acid domain (Gla), epidermal growth factor like domain (EGF), the activating peptide (AP) and the Serine Protease Domain, which is the FIX catalytic domain, are indicated. **b** Amino acid sequence of human FIX. The furin cleavage site is indicated and *symbols* above amino acid sequence indicate different post translational modifications; *open star* γ-carboxy glutamic acids, *filled star* β-hydroxylation, *open diamond* O-linked glycosylations, *filled diamond* N-linked glycosylations, *filled circle* sulfation, *open circle* phosphorylation. Peptide mapping by mass spectrometry of recombinant FIX protein showed a total sequence coverage of 94 % (*underlined*) when endoproteinase Lys-C, Arg-C and trypsin were used. The signal peptide amino acids are in lilac font and the propeptide amino acids are in *red* (Color figure online)

**Fig. 2:**
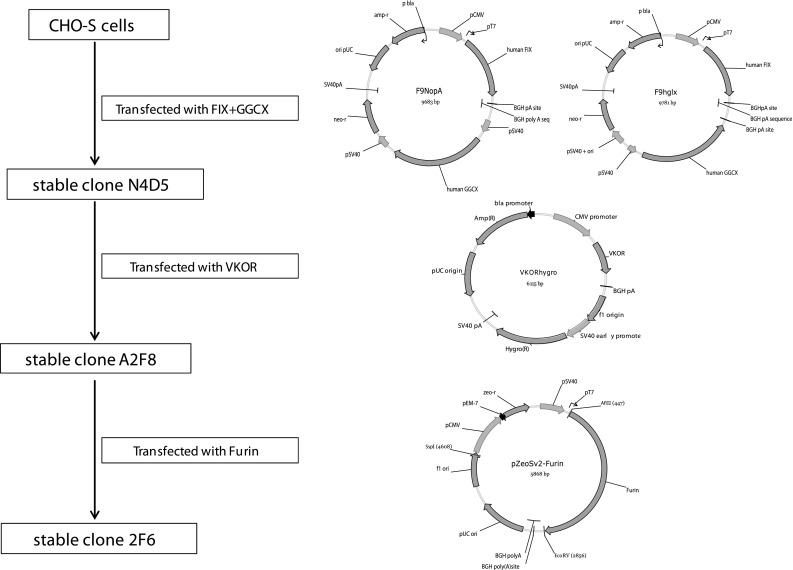
Overview of rhFIX cell line generation and maps of expression vectors. CHO-S cells were first transfected with the vectors F9NopA or F9hglx carrying FIX and GGCX cDNA. After screening of approximately 3,000 clones for FIX expression by a FIX-ELISA assay, the N4D5 clone originating from the F9NopA transfection was identified. This clone was then super-transfected with the vector VKORhygro carrying VKOR cDNA, and approximately 1,500 clones were screened for increased FIX activity. In the third step, the stable cell line A2F8 was super-transfected with the vector pZeoSV2-Furin carrying furin cDNA and approximately 600 clones were screened for increased FIX activity in order to identify the stable clone 2F6

Factor IX (FIX) is a protein undergoing many different post-translational modifications to be fully functional (Fig. 1a), including carboxylation of 12 glutamic acid residues in the N-terminal Gla domain. Achieving sufficient γ-carboxylation is one of the major challenges in high-tire production of recombinant human FIX (rhFIX). In this study, we have employed a new strategy for generating a production cell line for functional rhFIX. Our cell line development process involved three steps where modification enzymes were added to generate the required post-translational modifications. An overview of the FIX cell line development work is outlined in Fig. 2.

#### Co-expression of FIX with GGCX in CHO Cells

In the first step of our cell line development, we co-expressed FIX and GGCX. CHO-S cells were transfected with either F9NopA or F9hglx, two vectors carrying FIX and GGCX cDNA and engineered to obtain low levels of GGCX expression (Fig. 2). Analyses of the resulting clones showed that the F9NopA vector gave the most productive clones and also gave the lowest expression level of GGCX. From Real-Time RT-PCR analyses we found that, although human GGCX and rhFIX mRNA expression varied with culture time and conditions, the FIX:GGCX mRNA ratios were approximately the same for each clone. For the best rhFIX-producing clone N4D5 the ratio was approximately 45:1. Analyses of the best clone obtained with the F9hglx vector, P1G9, gave a lower ratio of approximately 4.5:1. This clone produced only 20 % of the amount rhFIX produced by N4D5 measured by FIX clot assay, even though the amount of rhFIX mRNA per cell was similar in these two clones.

Three rounds of shake flask expression experiments exploring different culture condition were made to evaluate growth characteristics as well as rhFIX expression of the chosen clones. Total rhFIX protein yield, including incompletely carboxylated and non-active rhFIX, was estimated by Western blotting to be at least 30 mg/L for the best clone, N4D5 (results not shown). The amount of active rhFIX was estimated by FIX clot assay to approximately 7 mg/L, which is a clear improvement compared to previously reported rhFIX expression levels obtained under comparable conditions which yielded approximately 1.5 mg/L active rhFIX [8]. Our results showed that controlled GGCX expression improved rhFIX productivity, although a large share of the rhFIX protein was still under-carboxylated.

#### Super-Transfection of Clone N4D5 with Vector Carrying VKOR cDNA

**Fig. 3:**
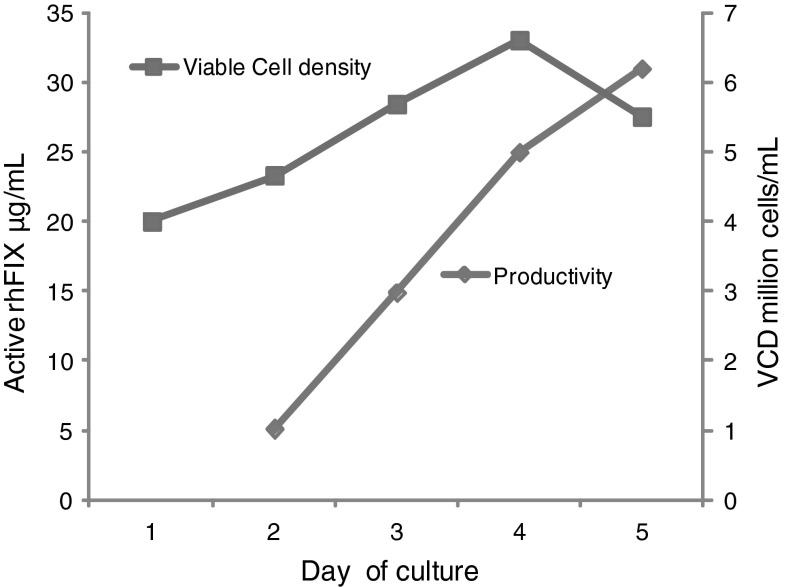
Recombinant human FIX production in shake flask culture. Cells were inoculated and cultivated as described in Sect. 2. FIX activity in the culture samples were determined by the ROX-FIX chromogenic FIX activity assay. Data are from one representative experiment with clone A2F8 grown at the most favourable conditions identified

To improve the share of active rhFIX the N4D5 clone was stably transfected with the linearized VKORhygro construct in which VKOR expression is controlled by the CMV promoter (Fig. 2). Obtained clones were screened for FIX productivity and VKOR expression was confirmed by RT-PCR (data not shown). Shake flask cultures from the best clone obtained, A2F8, had a total titer of rhFIX of 80–90 mg/L estimated by ELISA and produced up to 30 mg/L active rhFIX, determined by a chromogenic FIX activity assay (Fig. 3). The active rhFIX level obtained from A2F8 cells is significantly higher than previous clones (Table 1).

**Table 1:** Best rhFIX yields obtained during cell line development

Clone	Expression vectors	Active rhFIX (mg/L)	Culture condition
P1G9	F9hglx	1.3	Spinner bottle
N4D5	F9NopA	4	Small shake flask
N4D5	F9NopA	7.1	Spinner bottle
A2F8	F9NopA + VKORhygro	30	Shake flask

All cell cultures were performed at small scale (<100 mL) and FIX activity was measured by FIX clot assay (spinner bottles) or a chromogenic FIX assay (shake flasks) as described in Sect. 2

#### Super-Transfection of Clone A2F8 with Furin

Analysis of purified rhFIX protein from A2F8 cells showed that the propeptide of rhFIX was incompletely removed; about 30 % of the rhFIX still contained the propeptide. furin, also called PACE, is a protein that can proteolytically cleave propeptides, and has previously been reported to be involved in the processing of the FIX propeptide and found to improve FIX productivity in CHO cells [8]. To evaluate if processing of the rhFIX propeptide in clone A2F8 could be improved by co-expression of furin, the A2F8 cells were transfected with the linearized vector pZeoSv2-Furin DNA (Fig. 2), in which the furin cDNA is controlled by the SV40 promotor. After transfection and selection in Zeocin-containing medium, stable oligo-clonal pools were screened for improved rhFIX productivity using a chromogenic FIX activity assay (ROX-FIX). Clone 2F6 was found to have higher FIX activity. Recombinant hFIX from 2F6 shake flask cultures was partially purified via Q pseudo-affinity chromatography and subjected to N-terminal sequencing. Results showed that the propeptide of rhFIX was precisely and completely processed; showing that co-expression of furin improved propeptide processing of rhFIX.

### One-step Purification of Recombinant Human FIX

**Fig. 4:**
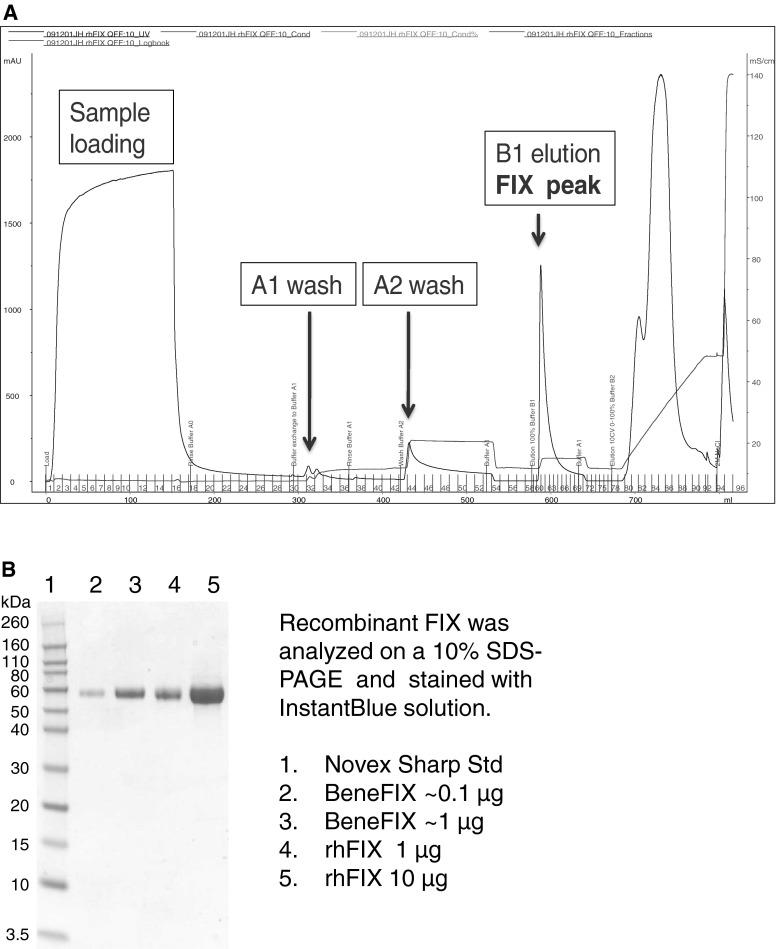
**a** QFF pseudo-affinity chromatography of rhFIX. The culture harvest (970 mL) was concentrated, then buffer exchanged to Buffer A0 (final volume 147 mL) and filtrated before loading onto a pre-equilibrated Q ion-exchange column. After washing with A1 and A2 buffers, the rhFIX protein was eluted by A3 buffer. **b** SDS-PAGE analysis of purified rhFIX protein. Purified and concentrated rhFIX was analysed together with Benefix on a 10 % SDS-PAGE under reducing condition. The gel was stained with InstantBlue solution. *Lane 1* Novex Sharp protein standard, *lane 2–3* Benefix, *lane 4–5* purified rhFIX

Different purification strategies for recombinant hFIX have been described in the literature, either using multi-steps of chromatography [9] or an affinity column with immobilized FIX antibody [10]. In this study, a simple and economical one-step purification method has been developed using ion-exchange chromatography (QFF). Recombinant FIX producing cells were grown in an in-house chemical defined serum-free medium with supplements (NEAA, GlutaMAX and Konakion^®^). After cultivation, cell culture medium was harvested, concentrated and purified as described in Sect. 2. During purification development, it was found that rhFIX bound relatively strong to the HiTrap Q column, at a salt concentration of 250 mM, rhFIX still remained on the column. The active rhFIX protein was eluted from the column with a buffer containing 100 mM NaCl and 20 mM CaCl_2_, which released rhFIX protein from the column via Ca^2+^-binding induced conformation change of FIX. The buffer used eluted little contaminating protein, and most of the under-carboxylated rhFIX remained on the column. An example of the chromatography is shown in Fig. 4a. The purity of rhFIX protein was estimated to be more than 90 % pure based on SDS-PAGE analysis (Fig. 4b) and the yield of rhFIX was 94 %.

Our results show that active recombinant FIX can be efficiently purified from cell culture media via a single-step AIEX chromatography method with a high yield.

### Characterization of Recombinant FIX

Human FIX protein consists of 415 amino acids with a theoretical molecular weight of 46,578 Da for the amino acid sequence alone, but its apparent MW is about 55 kDa due to numerous post-translational modifications. Recombinant human FIX was produced as a single chain zymogen. The purified rhFIX was analyzed on SDS-PAGE with an apparent molecular weight of 55 kDa (Fig. 4b), which is in good agreement with data reported previously [7]. Mass spectrometry analysis of rhFIX showed a molecular weight of about 54,770 Da. A broad peak was observed consistent with the complex post-translational modifications, for example different glycosylated forms (data not shown). Peptide mapping analysis of rhFIX showed a total sequence coverage of 94 % (underlined in Fig. 1b) when endoproteinase Lys-C, Arg-C and trypsin were used. Both N- and C-terminal peptides were identified in the analyses (Fig. 1b). Plasma derived FIX contains 12 γ-carboxylated glutamic acid residues in its N-terminal Gla domain, and the γ-carboxylation modification of these residues is critical for FIX’s maturation and function [11]. The content of γ-carboxylation of purified rhFIX was analyzed by MS. All observed γ-carboxylated forms for the N-terminus (amino acid 1–43) are listed in Table 2. The three dominant forms have 9, 10 and 11 γ-carboxyglutamic acids. About 70 % of the purified rhFIX contained 10 or more γ-carboxyglutamic acids. MS data also showed that Glu-40 was γ-carboxylated to a very low extent.

**Table 2:** Gamma-carboxylation analysis

No. of GLA	Percentage
12	5
11	26
10	36
9	22
8	7
7	3

There are 12 possible γ-carboxy glutamic acid residues (GLA) in total

## Discussion

Co-expression of modification enzymes to improve post-translational modifications of FIX has been attempted before with variable success. Co-expression of PACE (a secreted soluble form of furin) is utilised in the production of BeneFIX, and has been shown to improve productivity of FIX in CHO cells. On the other hand Hallgren et al. showed that either in the absence or presence of PACE expression, co-expression of GGCX together with FIX failed to improve secreted FIX specific activity [12, 13].

We believe that co-expression of modification enzymes need careful considerations for an optimal result. The intra-cellular location of modification enzymes and interaction with endogenous host cell machinery may require that the expression of added enzymes are restricted or controlled. With the work presented here, we have demonstrated that the productivity of rhFIX in mammalian cells can be dramatically improved by co-expressing enzymes to support γ-carboxylation and proteolytic processing. Our data suggest that controlled co-expression of GGCX gave the increase in total product titre. The level of GGCX expression giving the highest titre of FIX was surprisingly low; real-time PCR analyses indicated that less than 50 GGCX mRNA transcripts per cell were formed using the NopA expression vector, which gave the highest total rhFIX titre and an improved yield of active rhFIX.

The co-expression of VKOR improved γ-carboxylation but did not increase total rhFIX titre, which is in agreement with previously published data [14].We noted that the addition of VKOR improved growth properties of the FIX expressing clones, most notably by improving the survival of clones during the limiting dilution cloning procedures, and gave faster growth rates. The requirement of vitamin K addition to the culture medium decreased significantly and it was no longer necessary to feed extra vitamin K to the VKOR-transfected clones during rhFIX production. This suggests that the addition of VKOR has additional benefits in that vitamin K was efficiently recycled and accumulation of vitamin K epoxide, which could potentially be toxic to the host cells, was avoided.

Co-expression of furin significantly enhanced propeptide processing of FIX, which may affect specific activity. However, with the work reported here we were not able to demonstrate a clear impact on specific activity with or without the propeptide present. The fact that furin improved productivity of active FIX may just as well be attributable to a more efficient secretion of the protein. We have co-expressed the full-length form of furin, which is located in the ER and may therefore take part in improving the overall processing of rhFIX.

Characterization of rhFIX produced by our methods showed that rhFIX had the expected molecular weight and was γ-carboxylated to a high degree. In comparison with plasma derived FIX which is fully γ-carboxylated having 12 glutamic acid residues in the Gla domain and BeneFIX having 10 or more γ-carboxyglutamic acid residues, γ-carboxylation was lower in our FIX [7, 15]. However, earlier studies have shown that γ-carboxylation at Glu-36 and Glu-40 were not important for FIX function [16] suggesting that the γ-carboxylation of our rhFIX may be sufficient for high clotting activity. Further purification can also be done in order to remove under-carboxylated species. The first EGF domain of plasma derived FIX has three different post-translational modifications, two O-linked glycosylations on serine residues 53 and 61 [17, 18] and β-hydroxylation modification on aspartic acid at position 64 [19]. Mass spectometric studies showed that our rhFIX was β-hydroxylated on aspartic acid to about 40 % at position 64. This result is similar to what have been reported earlier for both plasma derived FIX and BeneFIX [15]. Also O-linked glycosylations in the first EGF domain of our rhFIX were studied by MS and showed that Ser-53 had a uniform O-glycosylation, Xyl-Xyl-Glc, whereas Ser-61 had three different O-linked forms, NeuAc-Gal-GlcNac-Fuc, Gal-GlcNAc-Fuc, and Fuc. These results are in line with what have been reported for other recombinant FIX produced [15, 20].

In summary, we hope that the expression strategies and results presented in this paper will contribute to the development of affordable coagulation factor products.

## Abbreviations

FIX: Factor IX
CHO: Chinese hamster ovary
GGCX: γ-Glutamyl carboxylase
Gla: γ-Carboxy glutamic acid
VKOR: Vitamin K epoxide reductase
Furin/PACE: Paired basic amino acid cleaving enzyme
PTMs: Post-translational modifications
SDS-PAGE: SDS-polyacrylamide gel electrophoresis
RT-PCR: Reverse transcription polymerase chain reaction
ELISA: Enzyme-linked immunosorbent assay
MS: Mass spectrometry
HPLC: High performance liquid chromatograhy
